# Amphipathic Peptide Antibiotics with Potent Activity against Multidrug-Resistant Pathogens

**DOI:** 10.3390/pharmaceutics13040438

**Published:** 2021-03-24

**Authors:** Jingru Shi, Chen Chen, Dejuan Wang, Ziwen Tong, Zhiqiang Wang, Yuan Liu

**Affiliations:** 1 College of Veterinary Medicine, Yangzhou University, Yangzhou 225009, China; shijr2021@gmail.com (J.S.); chenyzu19@gmail.com (C.C.); wangyzu2020@gmail.com (D.W.); tongzw2021@gmail.com (Z.T.); 2 Jiangsu Co-innovation Center for Prevention and Control of Important Animal Infectious Diseases and Zoonoses, Yangzhou University, Yangzhou 225009, China; 3 Joint International Research Laboratory of Agriculture and Agri-Product Safety, The Ministry of Education of China, Yangzhou University, Yangzhou 225009, China; 4 Institute of Comparative Medicine, Yangzhou University, Yangzhou 225009, China

**Keywords:** antimicrobial peptide, antibiotic resistance, multidrug-resistant bacteria, membrane damage

## Abstract

The emergence and prevalence of multidrug-resistant (MDR) bacteria have posed a serious threat to public health. Of particular concern are methicillin-resistant *Staphylococcus aureus* (MRSA) and *bla*_NDM_, *mcr-1* and *tet*(X)-positive Gram-negative pathogens. The fact that few new antibiotics have been approved in recent years exacerbates this global crisis, thus, new alternatives are urgently needed. Antimicrobial peptides (AMPs) originated from host defense peptides with a wide range of sources and multiple functions, are less prone to achieve resistance. All these characteristics laid the foundation for AMPs to become potential antibiotic candidates. In this study, we revealed that peptide WW307 displayed potent antibacterial and bactericidal activity against MDR bacteria, including MRSA and Gram-negative bacteria carrying *bla*_NDM-5_, *mcr-1* or *tet*(X4). In addition, WW307 exhibited great biofilm inhibition and eradication activity. Safety and stability experiments showed that WW307 had a strong resistance against various physiological conditions and displayed relatively low toxicity. Mechanistic experiments showed that WW307 resulted in membrane damage by selectively targeting bacterial membrane-specific components, including lipopolysaccharide (LPS), phosphatidylglycerol (PG), and cardiolipin (CL). Moreover, WW307 dissipated membrane potential and triggered the production of reactive oxygen species (ROS). Collectively, these results demonstrated that WW307 represents a promising candidate for combating MDR pathogens.

## 1. Introduction

The increasing emergence of multidrug resistance in pathogenic bacteria has posed a serious threat to public health. Of particularly concern are hard-to-treat Gram-negative bacteria [1], such as *Escherichia coli*, *Klebsiella pneumoniae*, *Pseudomonas aeruginosa*, and *Acinetobacter baumannii*, which were identified as a major cause of systemic infections in clinic [2]. In recent years, colistin is recognized as the last line of defense against multidrug-resistant (MDR) Gram-negative bacteria [3], while the emergence of mobile colistin resistance gene *mcr-1* has threatened the clinical effectiveness of colistin. Moreover, tigecycline resistance mediated by *tet*(X) and carbapenem resistance mediated by *bla*_NDM_ genes [4], and the co-harboring of *bla*_NDM_, *mcr-1* and/or *tet*(X) genes in clinical isolates leave no choice for clinicians in the treatment of MDR pathogens associated infectious diseases. Accordingly, if the development of drug resistance is not controlled, it may cause 10 million deaths by 2050, and the economic damage could exceed $100 trillion [5]. Nevertheless, few antibiotics have been approved for clinical use in the past decades [6], there is an urgent and unmet need to identify new antibiotic candidates [7].

Antimicrobial peptides (AMPs), also known as host defense peptides, have a wide range of sources and multiple functions such as protecting host from bacterial infections [8,9]. Compared with conventional antibiotics, bacteria are not prone to develop resistance against AMPs owing to its versatile mechanisms of action varies from membrane damage to interaction with intracellular targets [10,11,12]. Therefore, AMPs have been considered as a promising treatment option to combat the increasing drug-resistant pathogens [13]. Nevertheless, majority natural AMPs have not performed high optimization for direct antibacterial activity [14,15]. By contrast, synthetic AMPs by computer-assisted de novo design or modification from the sequences of naturally occurring AMPs are more effective against bacteria [16,17,18,19]. For example, a synthetic derivative of human AMPs LL-37, termed SAAP-148, exhibited improved bactericidal activity and plasma stability in killing MDR pathogens, including biofilm-producing bacteria and persister cells [20]. In addition, the truncation of MSI-78 at the *N*-terminal resulted in a 14-amino-acids AMPs named MSI-1 [21], which displayed superior activity and lower toxicity than its parent AMPs. Recently, two amphipathic peptide antibiotics termed horine and verine with systemic efficiency in mice infection models were identified [22]. Meanwhile, two optimized derivatives including WW304 and WW307 were proposed, which displayed lower hemolytic activity to red blood cells (RBCs) [22]. However, the antibacterial potential of these two peptides and its synergistic effect with clinically relevant antibiotics in the fight against MDR bacteria, particularly for hard-to-treat Gram-negative bacteria carrying *bla*_NDM_, *mcr*, and/or *tet*(X) genes, remains unknown.

Herein, we systematically evaluated the antimicrobial activity of WW304 and WW307 against a panel of MDR bacteria and investigated the potential mechanisms of action. Interestingly, WW307 could efficiently kill a panel of MDR pathogenic bacteria, including methicillin-resistant *Staphylococcus aureus* (MRSA) and MDR Gram-negative bacteria carrying *bla*_NDM_, *mcr*, and/or *tet*(X) genes. In addition, WW307 effectively prevented biofilm formation, and eradicated established biofilms and persister cells. Moreover, WW307 retained its activity under different physiological conditions in vitro. Our further experiments uncovered that the killing activity of WW307 is related to membrane permeabilization via the interaction with bacterial-specific phospholipids, the dissipation of membrane potential and the generation of ROS.

## 2. Materials and Methods

### 2.1. Peptide Synthesis and Validation

The peptides used in this paper were synthesized by solid phase peptide synthesis (SPPS) by GL Biochem (Shanghai, China), and their accurate molecular weights were determined by matrix-assisted laser desorption/ionization time-of-flight mass spectrometry. The purity of all peptides was greater than 95%, indicating that they were accurately obtained.

The chemical structure of the peptides was mapped using Chemdraw software. Helical wheel projection and various chemical parameters such as net charge and hydrophobicity were calculated with https://www.donarmstrong.com/cgi-bin/wheel.pl (accessed on 23 March 2021).

### 2.2. Antibacterial Activity Tests

#### 2.2.1. Minimum Inhibitory Concentrations (MICs) Determination

The MICs of all drugs were determined by micro-broth dilution method according to CLSI 2018 guidelines [23]. The bacterial suspension used in the experiment was appropriate 1.5 × 10^6^ colony-forming units (CFUs) per mL and mixed with the drug solution in a sterilized 96-well microtiter plate (Corning, New York, USA), then incubated at 37 °C for 16–18 h. MIC value was defined as the minimum drug concentration without visible bacteria. Meanwhile, the density of the bacteria at 600 nm was measured, and the corresponding IC_50_ was calculated using log (inhibitor) vs. response variable slope (four parameters) in Graphpad Prism version 8.3.0 software (San Diego, CA, USA). To assess the role of ROS production in the antibacterial activity of WW307, increasing concentrations of *N*-acetylcysteine (NAC) from 0 to 5 mM were added in culture medium, followed by MICs test.

#### 2.2.2. Mutant Prevention Concentrations (MPCs) Determination

For the MPC assay, 100 μL MRSA T144 or *E. coli* B2 suspensions (~10^10^ CFUs) were plated on Mueller-Hinton agar (MHA) plates containing increasing concentrations of WW307. Subsequently, the plates were incubated at 37 °C for 72 h. MPC was defined as the lowest peptide concentration that prevented the growth of resistant colonies [24]. For each strain, MPC was determined in at least two independent experiments.

#### 2.2.3. Salts and Serum Stability

In order to evaluate the influence of ions and serum on the activity of AMPs, 10 mM Na^+^, K^+^, Mg^2+^, 10% Dulbecco’s Modified Eagle Medium (DMEM) and fetal bovine serum (FBS) were added into Mueller-Hinton broth (MHB) culture medium for subsequent MIC tests.

#### 2.2.4. Thermal, pH and Proteolytic Stability

Drugs were incubated at different temperatures (range from 40 to 121 °C) and pH (range from 2 to 12) for 1 h, then adjust back to original pH, following MIC test to evaluate their residual activity [25]. To evaluate the protease stability, pepsin, trypsin, and papain were mixed with WW307 to achieve a final concentration of 1 mM, and then incubated at 37 °C for 1 h. After incubation, the remaining protease was precipitated using acetonitrile and removed by centrifugation at 3000× *g*, following for MIC test.

#### 2.2.5. LPS and Lipids Inhibition Assay

The effects of LPS and phospholipids (Sigma-Aldrich), including phosphatidylcholine (PC), phosphatidylethanolamine (PE), phosphatidylglycerol (PG), and cardiolipin (CL), on the antibacterial activity of WW307 were evaluated using the checkerboard microdilution assay [26]. Briefly, LPS from *E. coli* O111:B4 (0–128 μg/mL) and phospholipids (0–16 μg/mL) were 2-fold diluted into the broth, then bacteria suspension and WW307 mixture was added. Following MIC test to evaluate the activity changes of WW307.

### 2.3. Checkerboard Assays

The synergistic antibacterial activity of all peptides with different antibiotics were determined by micro-broth dilution using flat bottom 96-well microtiter plate (Corning, New York, USA). The mixture of bacterial solution, peptides and antibiotics were incubated at 37 °C for 16–18 h, and then the optical density (OD) at 600 nm was measured. Two biological replicates were performed for each combination and the corresponding FIC index (FICI) was calculated using the formula as follows [27]: ΣFIC = FICA + FICB; FICA = (MICA in the presence of B/MICA alone); FICB = (MICB in the presence of A/MICB alone). Synergy is defined as an FIC index of ≤0.5.

### 2.4. Time-Dependent Killing

MRSA T144 and *E. coli* B2 were cultured in 37 °C for 4–6 h, then diluted 1/1000 in MHB. In 96-well flat-bottom plates (Corning), different concentrations of WW307 (0–256 μg/mL) were added and mixed with bacterial suspension (~10^6^ CFUs/mL), then incubated at 37 °C for 0.5, 1, 2 and 4 h, respectively. At each time point, 20 μL of the above mixture was removed and mixed with 180 μL PBS. Subsequently, ten-fold serially diluted suspensions were plated on MHA plates and incubated overnight at 37 °C. The bacterial colonies were counted and the primary CFUs/mL was calculated.

### 2.5. Prevention of Biofilm Formation

The ability of WW307 on the prevention of biofilm formation was assessed using crystal violet method [28]. In brief, MRSA T144 and *E. coli* B2 (1.5 × 10^6^ CFUs/mL) mixed with different concentrations of WW307 were cultured in 96-well microtiter flat plate (Corning) at 37 °C. After 48 h incubation, the planktonic bacteria were removed by washing three times with sterile PBS solution. Afterward, 100 μL methanol was added and fixed for 15 min. Next, the fixed solution was sucked out for natural air drying. Dried wells were stained with 100 μL of 0.1% crystal violet for 15 min and the remaining crystal violet was rinsed with PBS for twice. Finally, 100 μL 33% acetic acid was added and cultured at 37 °C for 30 min to dissolve crystal violet. The absorbance at 570 nm was determined as a measure of biofilm mass.

### 2.6. Eradication of Established Mature Biofilms

Exponential phase MRSA T144 and *E. coli* B2 cells were diluted in 1/1,000, then mixed with 200 μL MHB and cultured in 96-well microtiter plate (Corning) at 37 °C for 48 h to promote biofilm formation [28]. After washed three times with PBS, different concentrations of WW307 were added and cultured at 37 °C. After 2 h of incubation, the wells were emptied, washed, and sonicated for 15 min to disperse biofilm cells. Next, bacteria diluted suspensions were plated on MHA plates and incubated overnight at 37 °C. Bacterial colonies were counted and the primary CFUs per mL were calculated. Finally, the remaining CFUs were used to evaluate the removal of the biofilm.

### 2.7. Hemolysis Analysis

The hemolytic activity on all peptides was evaluated based on previous report [28]. Briefly, fresh Sheep RBCs were washed twice with phosphate buffer (PBS), and then 8% red blood cell suspension was prepared. The increasing concentrations of AMPs and melittin were mixed with 8% red blood cell suspension, respectively, and incubated at 37 °C for 1 h. The sterilized PBS and double-distilled water (ddH_2_O) were used as blank and positive control, respectively. Afterwards, the supernatant was centrifuged to measure the absorption of released hemoglobin at 576 nm by an Infinite M200 Microplate reader (Tecan, Männedorf, Switzerland). The corresponding hemolysis rate was calculated.

### 2.8. Circular Dichroism (CD) Measurements

CD spectra of the peptides in four different solvents, including 0.01 M PBS (pH = 7.2), 50 μM LPS, 50 mM sodium dodecyl sulfate (SDS), and 50% trifluoroethanol (TFEA) [21], were measured using a J-810 spectropolarimeter (Jasco, Tokyo, Japan) at 25 °C. The wavelength recorded values were 190–300 nm, and the measurements were repeated three times.

### 2.9. Outer Membrane Permeabilization

Overnight MRSA T144 and *E. coli* B2 cells were washed and resuspended in PBS to obtain an OD_600_ of 0.5, followed by the addition of 0.1 μM of 1-*N*-phenylnaphthylamine (NPN) (Aladdin), and incubated in a constant temperature shaking table at 37 °C for 30 min in dark [29]. Subsequently, 190 µL of probe-labelled cells were incubated with 10 µL of WW307 (0 to 128 µg/mL) in a sterile 96-well black plate for 1 h. After incubation, the fluorescence intensity (λexcitation = 350 nm, λemission = 420 nm) was measured using an Infinite M200 Microplate reader (Tecan, Männedorf, Switzerland).

### 2.10. Membrane Permeability Assay

The fluorescent dye propidium iodide (PI) (Beyotime, Shanghai, China) was used to assess the integrity of bacterial cell membranes [30]. MRSA T144 and *E. coli* B2 cells were incubated with PI (0.5 μM) for 30 min in dark, and the labelled cells were treated with increasing concentrations of WW307. After 1 h incubation, the fluorescence intensity (λexcitation = 535 nm, λemission = 615 nm) was determined using an Infinite M200 Microplate reader (Tecan, Männedorf, Switzerland).

### 2.11. Cytoplasmic Membrane Potential

3′, 3′-dipropylthiadicarbocyanine iodide (DiSC_3_(5)) (Aladdin, Shanghai, China) was applied to determine the membrane potential of MRSA T144 and *E. coli* B2 [30]. MRSA T144 and *E. coli* B2 suspensions (OD_600_ = 0.5) were incubated with DiSC_3_(5) (0.5 μM) in dark for 30 min and incubated with WW307 (0–128 μg/mL) for 1 h. Then, the fluorescence intensity (λexcitation = 622 nm, λemission = 670 nm) was determined using an Infinite M200 Microplate reader (Tecan, Männedorf, Switzerland).

### 2.12. ROS Measurements

2′,7′-dichlorodihydrofluorescein diacetate (DCFH-DA) (Beyotime) was applied to monitor the levels of ROS in MRSA T144 and *E. coli* B2. DCFH-DA (10 μM) was pre-incubated with MRSA T144 and *E. coli* B2 cells for 30 min, then removed excess fluorescent probes that have not entered the cells by centrifugation and washing with PBS. Subsequently, the probed-cells were incubated with varying concentrations of WW307 (0–128 μg/mL) for 1 h in dark. After incubation, the fluorescence intensity (λexcitation = 488 nm, λemission = 525 nm) was immediately measured using an Infinite M200 Microplate reader (Tecan, Männedorf, Switzerland).

### 2.13. Statistical Analysis

All data were shown as mean ± SD. Statistical significance was determined by unpaired *t* test between two groups or non-parametric one-way ANOVA among multiple groups using GraphPad Prism 8 (NS, not significant; * *p* < 0.05, ** *p* < 0.01, *** *p* < 0.001).

## 3. Results

### 3.1. Characterizations of Peptides

**Figure 1:**
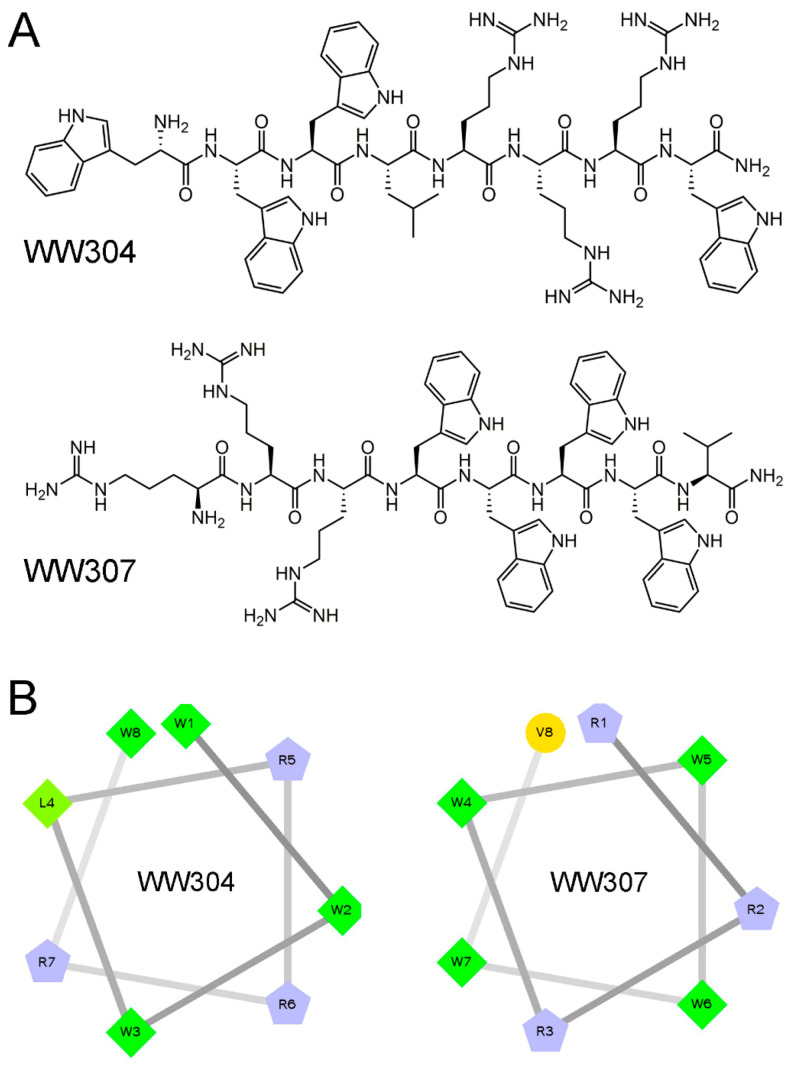
Design and characterization of two amphipathic Antimicrobial peptides (AMPs). (**A**) Chemical structures of two active AMPs (WW304 and WW307). (**B**) Helical wheel projections of WW304 and WW307. The hydrophilic residues as circles, hydrophobic residues as diamonds, and potentially positively charged as pentagons. Hydrophobicity is color coded as well: the most hydrophobic residue is green, and the amount of green is decreasing proportionally to the hydrophobicity, with zero hydrophobicity coded as yellow.

As shown in Table 1, WW304 and WW307 were all cationic peptides, with net charges of +3. The purity of the two peptides were greater than 95%, indicated that the peptides were obtained accurately. The wheel diagram showed both WW304 and WW307 exhibited imperfect amphiphilic structures that possessed interrupted hydrophobic and cationic faces (Figure 1).

### 3.2. Potent Antibacterial Activity of Peptide In Vitro

**Figure 2:**
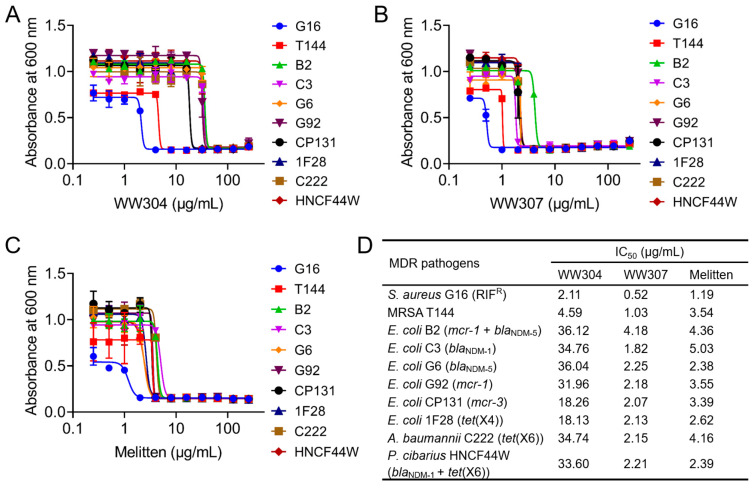
Half-maximal inhibitory concentrations (IC_50_) assay of WW304, WW307, and melitten against 10 tested MDR pathogens. Dose response curves (**A**–**C**) and IC_50_ values (**D**) of WW304, WW307, and melitten on tested pathogens.

Next, we tested the antimicrobial activity of WW304 and WW307 against a panel of MDR bacteria, including MRSA and Gram-negative bacteria co-carrying *bla*_NDM_, *mcr*, and/or *tet*(X) genes. Results showed that WW307 displayed the best activity against all test MDR bacteria with MIC values from 1–8 μg/mL (Table 2), while WW304 only had modest activity against Gram-positive bacteria (MIC, 8 μg/mL) rather than Gram-negative bacteria (MIC, 32 or 64 μg/mL). In addition, the MPC of WW307 against MRSA T144 and *E. coli* B2 was 64 μg/mL. Meanwhile, we determined the IC_50_ values of WW304 and WW307 against all tested strains using melittin as a control. In agreement with the MIC analysis, WW307 showed the lowest IC_50_ values from 0.52 to 4.18 μg/mL, indicating that WW307 had superior antibacterial activity than WW304 and melittin (Figure 2). The potent broad-spectrum activity of WW307 against these MDR pathogens implied that the activity of WW307 was independent of the current existing resistance determinants. Therefore, WW307 was selected as a drug candidate for our following studies.

### 3.3. WW307 Is a Potent Antibiotic Adjuvant

**Figure 3:**
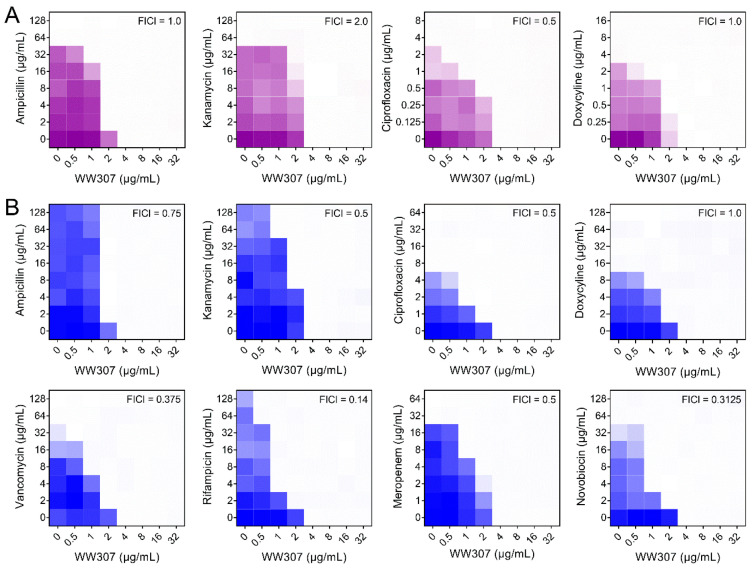
Checkerboard broth microdilution assays between WW307 with different classes of antibiotics against methicillin-resistant *Staphylococcus* (MRSA) T144 and *E. coli* B2. (**A**) Synergistic antibacterial activity of WW307 with four antibiotics against MRSA T144. (**B**) Synergistic antibacterial activity of WW307 with eight antibiotics against *E. coli* B2. Bacterial cells and drugs were cultured at 37 °C for 16–18 h, then the density of cells at 600 nm was measured, the darker color means the higher density of bacteria. Data represent the mean of two biological replicates.

The increasing problem of antibiotic resistance calls for new therapeutic strategies, such as antibiotic adjuvants, which offer a productive approach to combat MDR pathogens [31]. For example, cationic AMPs were found to synergize with azithromycin against MDR Gram-negative bacteria [32,33]. As such, we evaluated the adjuvant potency of WW307 in combination with different classes of antibiotic via checkerboard assay using two MDR isolates MRSA T144 [34] and *E. coli* B2 [35] as test strains. Consequently, we found that WW307 showed no synergistic activity against MRSA T144 when in combination with different antibiotics (Figure 3A), while WW307 potentiated the rifampicin, novobiocin and vancomycin activities against *bla*_NDM-5_ and *mcr-1* co-carrying *E. coli* B2 (Figure 3B), with FICI of 0.14, 0.3125 and 0.375, respectively. Interestingly, WW307 had synergistic antibacterial activity with hydrophobic antibiotics such as rifampicin, novobiocin and vancomycin against *E. coli* B2, rather than ampicillin, kanamycin, ciprofloxacin, doxycycline, and meropenem. These results suggested that WW307 effectively synergized with Gram-positive active antibiotics against Gram-negative bacteria. Considering that the outer membrane of Gram-negative bacteria serves as a barrier for Gram-positive active antibiotics, we speculated that WW307 may serve as an outer membrane-active targeted compounds.

**Figure 4:**
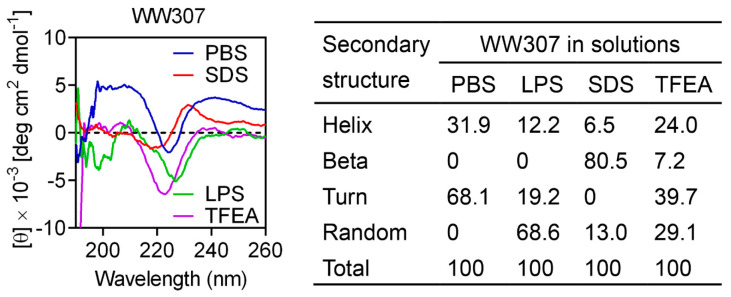
Circular dichroism (CD) spectra of WW307 in various solutions. PBS (10 mM, pH = 7.4), lipopolysaccharide (LPS) (50 μM), sodium dodecyl sulfate (SDS) (50 mM), and 50% TFEA were used. The values from three scans were averaged per sample, and the peptide concentrations were fixed at 100 μg/mL.

Considering the potential antibacterial activity and synergistic antibacterial activity of WW307, we further determined the secondary structure of WW307 in different solvents by CD analysis. As LPS is an important component of the outer membrane of Gram-negative bacteria [36], thus LPS was chosen to simulate the bacterial cell environment. The SDS with negative charge surface and TFEA were used to simulate the anionic membrane environment and hydrophobic environment of bacterial membrane, respectively [37]. As shown in Figure 4, WW307 exhibited helix and turn mixed structure in PBS. In 50 μM LPS, 50 mM SDS, and 50% TFEA, the helix ratio showed a downward trend and the proportion of random increased, while in SDS, the ratio of beta sheet increased to 80.5%. All this implied that secondary structures of WW307 would present in a hybrid form when it interacts with the bacterial membrane.

### 3.4. Rapid Bactericidal Efficiency of WW307 against MDR In Vitro

**Figure 5:**
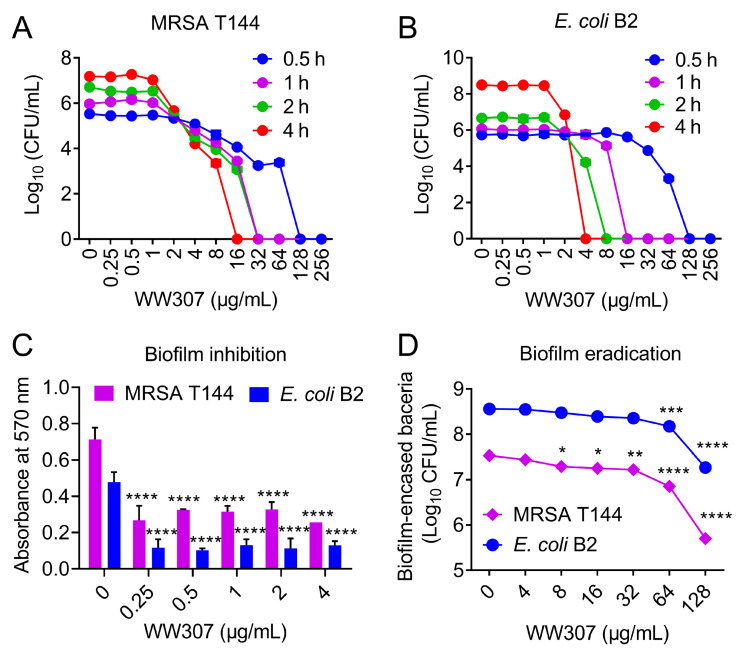
Bactericidal ability and anti-biofilm activity of WW307. (**A**,**B**) The time- and concentration-killing curves of WW307 against MRSA T144 and *E. coli* B2. The mixture of varying concentrations of WW307 (0–256 μg/mL) and bacterial solution was incubated at 37 °C for 0.5, 1, 2, and 4 h, respectively. Afterwards, ten-fold serially suspensions were plated on MHA plates and incubated overnight, and the corresponding CFUs were counted and calculated. (**C**) Inhibitory effects of WW307 on MRSA T144 and *E. coli* B2 biofilm formation. Bacterial cells were cultured in 96-well plates at 37 °C with or without WW307 (0–4 μg/mL). After 48 h incubation, the biofilm mass was determined using crystal violet method. (**D**) Eradication abilities of WW307 against mature biofilm by MRSA T144 and *E. coli* B2. Bacterial suspensions were cultured at 37 °C for 48 h. Then, WW307 from 0 to 128 μg/mL was added and incubated at 37 °C for 2 h. Eradication effect of WW307 on biofilm was evaluated by determining the remaining CFUs. All data from at least three biological replicates were presented as mean ± SD, and the statistical significance was determined by unpaired *t* test (**C**) or non-parametric one-way ANOVA (D) (* *p* < 0.05, ** *p* < 0.01. *** *p* < 0.001, **** *p* < 0.0001).

In consideration of the potent growth inhibitory activity of WW307 on MDR pathogens, so we next performed time- and concentration-dependent killing assays [38] to determine whether WW307 has a great bactericidal activity. As shown in Figure 5A,B, WW307 exhibited rapid killing effect on MRSA T144 and *E. coli* B2, and WW307 can completely eradicate MRSA T144 and *E. coli* B2 at 32 μg/mL and 16 μg/mL during one hour, respectively. Moreover, the bactericidal activity of WW307 exhibited an obvious time- and concentration-dependent manner. These results suggested that WW307 was a potent bactericidal antibiotic.

### 3.5. WW307 Exhibits Biofilm Inhibition and Eradication Activities

The production of biofilms by bacteria makes it difficult for common antibiotics to function and easy to cause chronic infections [28]. Therefore, we further evaluated the ability of WW307 on biofilms formation and eradication. As shown in Figure 5C, we observed that WW307 dose-dependently inhibited the formation of biofilms by MRSA T144 and *E. coli* B2 using crystal violet assay. Excitingly, a significant inhibition effect was also observed under the treatment of low level of WW307 (0.25 μg/mL, corresponding to 0.0625-fold MIC). However, the crystal violet staining method cannot characterize the effect of WW307 on metabolic activity in biofilm. The use of other methods such as 2,3,5-triphenyl-tetrazolium chloride (TTC) analysis [39] would strengthen the inhibitory ability of WW307 on biofilm formation. Furthermore, we found that established biofilms of MRSA T144 and *E. coli* B2 formed on a plasma-coated surface were remarkably eradicated by WW307 in a dose-dependent manner (Figure 5D).

### 3.6. A Desirable Stability and Safety of WW307 against Bacteria

The great stability of AMPs is a critical prerequisite for its in vivo efficacy [40]. Therefore, we assessed the residual antibacterial activity of WW307 after exposure to different temperatures and pH conditions [21]. Surprisingly, WW307 completely retained its activity after treatment under 40–121 °C or pH (2 to 12) for 1 h, indicating that WW307 possessed great thermal and pH stability (Table 3). We speculated that the great stability of WW307 may be related to the unspecific or mixed secondary structures of WW307 in solutions, which have been evidenced by CD assay. Next, we evaluated the activity of WW307 in three salt ions including Na^+^, K^+^ and Mg^2+^ [41]. As shown in Table 3, WW307 retained full activity against MRSA T144 and *E. coli* B2 in the presence of monovalent cation, while divalent cation mg^2+^ remarkably reduced the activity of WW307. Given that the divalent cation is usually as the stabilizer of outer membrane of Gram-negative bacteria [42], it can be speculated that the action of WW307 may be related to bacterial membrane damage. In addition, culture media containing 10% serum and DMEM were used to simulate in vivo matrix environment. The results showed that the antibacterial activity of WW307 in these two substrates reduced only two folds. Moreover, the proteolytic stability of WW307 after incubated with different proteases, including pepsin, trypsin and papain, was evaluated. No loss of antibacterial activity of WW307 in the presence of pepsin was found, whereas trypsin and papain completely abolished the activity of WW307. This may correlate with the amino acid composition of WW307, especially the high proportion of arginine at the *N*-terminal [43].

**Figure 6:**
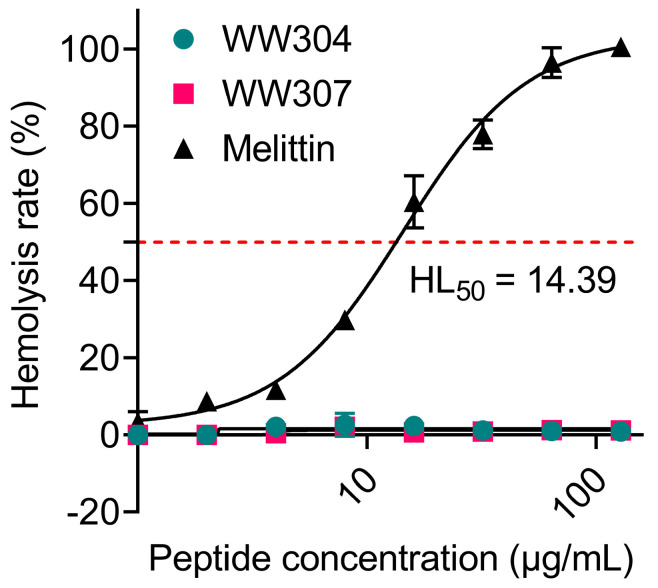
Hemolytic activity of peptides against mammalian red blood cells (RBCs). HL_50_ is the peptide concentration that causes 50% hemolysis. Sterilized PBS and ddH_2_O were used as a negative control and positive control, respectively.

Safety is a key factor that prevents AMPs from entering clinical use, thus we further evaluated the hemolytic properties of WW307 with defibrillated sheep red blood cells. Consistent with previous study [22], Figure 6 showed that WW304 and WW307 had the dispensable hemolytic activity (HL_50_ > 128 µg/mL) on mammalian RBCs, while melittin exhibited high hemolytic activity with HL_50_ of 14.39 µg/mL, indicating that WW307 had higher selectivity for bacteria rather than mammalian cells.

### 3.7. WW307 Targets Bacteria Membrane Components and Leads to Membrane Damage

**Figure 7:**
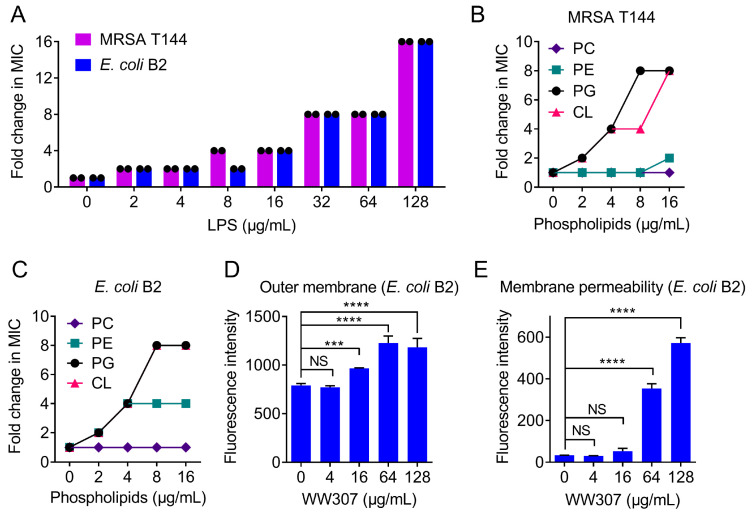
WW307 disrupts bacterial membrane permeability by specifically targeting lipopolysaccharide (LPS) and various phospholipids. (**A**) Exogenous addition of LPS deriving from *E. coli* O111:B4 impaired the antibacterial activity of WW307 against MRSA T144 and *E. coli* B2 in a dose-dependent manner. (**B**,**C**) Increased MICs of WW307 against MRSA T144 and *E. coli* B2 in the presence of PG, CL and PE. The 96-well plates were incubated at 37 °C for 16-18 h for MIC analysis. (**D**,**E**) WW307 disrupted the outer membrane and whole cell membrane permeability of *E. coli* B2, which were assessed by fluorescence probes 1-*N*-phenylnaphthylamine (NPN, excitation 350 nm and emission 420 nm) and propidium iodide (PI, excitation 535 nm and emission 615), respectively. Bacterial cells were incubated with fluorescent probes at 37 °C for 30 min in dark, then different concentrations of WW307 (0–128 μg/mL) was added and incubated at 37 °C for 1 h. All data were presented as mean ± SD, and the statistical significance was determined by non-parametric one-way ANOVA (NS, not significant; *** *p* < 0.001, **** *p* < 0.0001).

Having shown the potent antibacterial and bactericidal activities of WW307, we next sought to elucidate the potential mechanisms of action of WW307. The synergistic antibacterial activity indicated that WW307 could significantly promote the antibacterial activity of Gram-positive active antibiotics against *E. coli* B2, suggesting that WW307 might be a membrane-active antibiotic. Moreover, the ionic stability indicated that divalent cation Mg^2+^ supplementation could significantly impair the antibacterial activity of WW307 (Table 3). Considering that LPS locates in outer membrane of Gram-negative bacteria are commonly stabilized with divalent cations particularly Mg^2+^ [44], therefore, we further speculated that WW307 might compete with Mg^2+^ for binding to LPS. All these points implied that WW307 is an outer membrane destructive peptide antibiotic. In addition to the outer membrane, cytoplasmic membrane is an important barrier in both Gram-positive and Gram-negative bacteria. Accordingly, PG, CL and PE are the main constituents of the cytoplasmic membrane, while PC mainly exists in eukaryotic cells [45]. To investigate whether WW307 could target these components of bacterial membrane, varying concentrations of exogenous LPS, PG, CL, PE and PC were added in MIC analysis. The results showed that exogenous addition of LPS abolished the antibacterial activity of WW307 against MRSA T144 and *E. coli* B2 (Figure 7A), suggesting the close interaction of WW307 and LPS. In addition to LPS, PG and CL supplementation except for PC remarkably impaired the antibacterial activity of WW307 (Figure 7B,C), indicating a great selectivity of WW307. These results suggested that WW307 could specifically bind to bacterial membrane related components, such as LPS, PG and CL.

To further characterize membrane damage caused by WW307, a hydrophobic fluorescence probe 1-*N*-phenylnaphthylamine (NPN) [30] was applied to evaluate the effect of WW307 on the outer membrane permeability of *E. coli* B2. As a result, the fluorescence intensity showed a concentration-dependent increase, indicating that WW307 seriously disrupted the outer membrane of *E. coli* B2 (Figure 7D). Next, we used propidium iodide (PI) to evaluate the whole membrane permeability [46]. As shown in Figure 7E, WW307 led to a dose-dependent increase of PI fluorescence in *E. coli* B2, implying a remarkable damage to the bacterial membrane. Taken together, all above results suggested that WW307 was a membrane destructive antibiotic through targeting bacteria membrane related components.

### 3.8. WW307 Dissipates the ΔΨ Component of Bacterial Proton Motive Force

**Figure 8:**
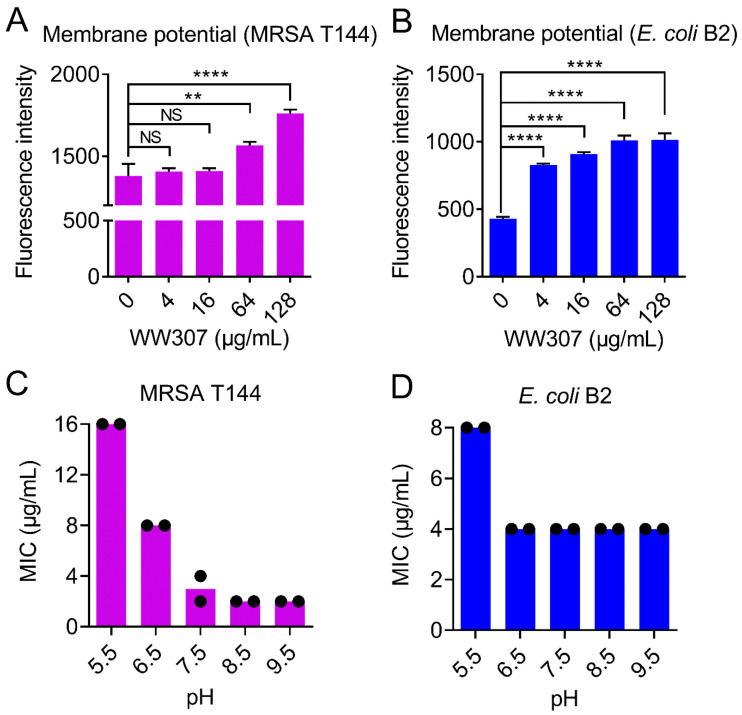
WW307 dissipates the ΔΨ component of the proton motive force (PMF). (**A**,**B**) WW307 dissipated the membrane potential of MRSA T144 and *E. coli* B2, determined by monitoring the fluorescence intensity of 3,3’-dipropylthiadicarbocyanine iodide (DiSC_3_(5), excitation at 622 nm and emission at 670 nm). DiSC_3_(5)-labeled cells were incubated with WW307 at 37 °C for 1 h prior to fluorescence intensity determination. (**C**,**D**) The shift to alkaline environment enhanced the antibacterial activity of WW307 against MRSA T144 and *E. coli* B2. In A and B, all data were expressed as mean ± SD, and the statistical significance was determined by non-parametric one-way ANOVA (NS, not significant; ** *p* < 0.01, **** *p* < 0.0001).

Proton motive force (PMF) is generated by bacterial transmembrane potential and is made up of two parameters: the electric potential (ΔΨ) and the transmembrane proton gradient (ΔpH). These two components work together to maintain dynamic balance of the PMF, which is critical to bacterial survival [47]. To investigate the influence of WW307 on the membrane potential of MRSA T144 and *E. coli* B2, 3′,3′-dipropylthiacarbocyanine iodide (DiSC_3_(5)) was applied for this purpose [35]. DiSC_3_(5) accumulates in the cytoplasmic membrane in response to the ΔΨ component of the PMF. As the disruption of ΔΨ, the probe would be released into the extracellular milieu and result in increased fluorescence. Conversely, the disruption of ΔpH would be compensated by increasing ΔΨ, resulting in enhanced DiSC_3_(5) uptake into the cytoplasmic membrane and therefore decreased fluorescence [48]. Our results showed that exposure of cells into varying concentrations of WW307 resulted in an immediate increase in DiSC_3_(5) fluorescence in a dose-dependent manner (Figure 8A,B), suggesting that WW307 selectively dissipated the ΔΨ component of the PMF. To validate this finding, we next assay the MIC changes of WW307 in different pH nutrient matrix conditions. As shown in Figure 8C,D, we observed that the antibacterial activity of WW307 decreased under acidic conditions, but increased under alkaline conditions, which is consistent with previous results that WW307 disrupted the membrane potential of MRSA T144 and *E. coli* B2.

### 3.9. WW307 Promotes the Production of ROS

**Figure 9:**
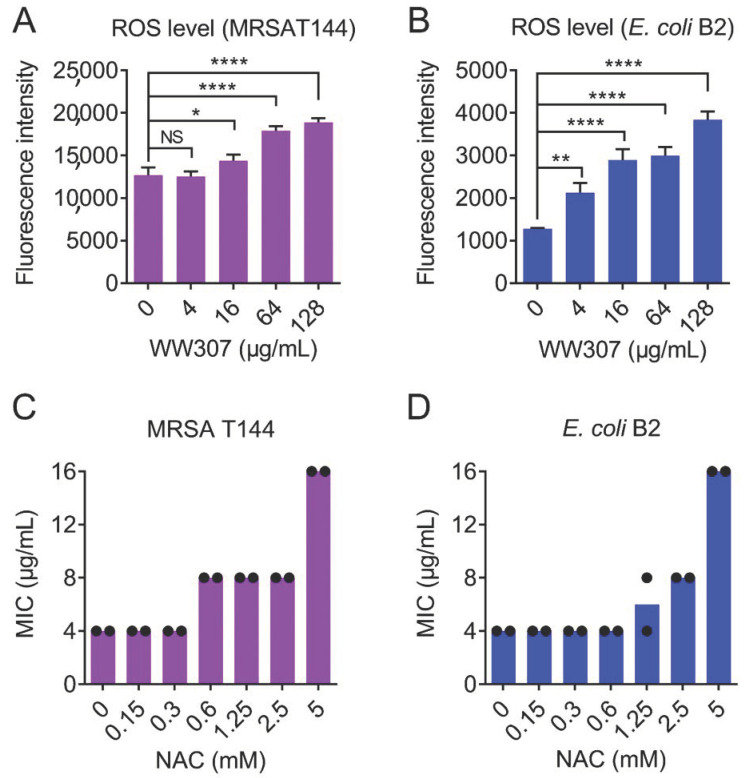
WW307 triggers the production of ROS in MRSA T144 and *E. coli* B2. The production of ROS of MRSA T144 (**A**) and *E. coli* B2 (**B**) was determined by 2’,7’-dichlorodihydrofluorescein diacetate (DCFH-DA, excitation at 488 nm and emission at 525 nm). Before fluorescence assay, probe-labelled cells were incubated with WW307 at 37 °C for 1 h. (**C**,**D**) *N*-acetylcysteine (NAC) supplementation abolished the antibacterial activity of WW307 against MRSA T144 and *E. coli* B2. In A and B, all data were presented as mean ± SD, and the statistical significance was determined by non-parametric one-way ANOVA (NS, not significant; * *p* < 0.05, ** *p* < 0.01, **** *p* < 0.0001).

The production of reactive oxygen species (ROS) has been recognized as a key factor for antibiotic-mediated killing. Given that WW307 is a potential bactericidal peptide antibiotic, we next used 2′,7′-Dichlorodihydrofluorescein diacetate (DCFH-DA) [49] to measure the ROS level in bacteria imposed by WW307. Consistently, WW307 triggered the production of ROS in a concentration-dependent manner (Figure 9A,B). To evaluate the role of ROS generation in the antibacterial activity of WW307, ROS scavenger NAC was added in the following MIC analysis. Interestingly, addition of NAC (5 mM) dramatically abolished the activity of WW307 against MRSA T144 and *E. coli* B2, with 4-fold increase of MIC values (Figure 9C,D). These results suggested that the production of ROS is essential for the bactericidal activity of WW307 against MDR bacterial pathogens.

## 4. Discussion

Recalcitrant and complicated infections in clinics caused by MDR pathogens, particularly for Gram-negative bacteria, call for novel antibacterial pipeline. Owing to the limited choice from existing antibiotic regimens in the resistance era, AMPs have attracted global attention as novel drug candidates for the treatment of infectious diseases. In this study, we investigated the antibacterial activity and mechanisms of action of two amphipathic peptides (WW304 and 307) in the fight against MDR pathogens. As a consequence, we found that WW307 exhibited potent broad-spectrum bacteriostatic and bactericidal activity for all tested pathogens, including emerging *bla*_NDM_, *mcr* and/or *tet*(X)-positive Gram-negative bacteria. Most importantly, the stability and safety evaluation indicated that WW307 retained its activity under various physiological conditions and displayed no hemolytic activity below 128 μg/mL. Nevertheless, more preclinical studies, including in vivo efficacy and toxicity evaluation in mammals, are required to further verify its effectiveness and safety.

With regard to the modes of action of WW307, we found that WW307 is a membrane-active peptide antibiotic. Consistently, most AMPs particularly for cationic AMPs exert bactericidal effects by disrupting the membrane integrity [50]. Heretofore, three classical models, including barrel-stave model, toroidal-pore model, and carpet model, were proposed to describe membrane perturbation by AMPs [51]. For example, amphibian-derived AMPs magainin 2 [52] and aurein 1.2 [53] disrupted membrane via the toroidal-pore and carpet mechanisms, respectively. In our studies, we further revealed that WW307 disrupted membrane permeability by specifically binding to bacterial membrane-specific components, including LPS, PG, and CL. Considering that these targets carry negatively charged head groups, we proposed electrostatic force between positively charged WW307 and these targets may account for its membrane interaction. Additionally, we also found that WW307 dissipated the ΔΨ component of bacterial PMF, which is essential for a variety of critical bacterial processes, such as ATP synthesis, flagellar motility, and nutrient import [47]. Recently, a novel antibiotic named halicin was identified using a deep learning approach [54]. Notably, halicin acts by dissipates the ΔpH component of PMF. These examples implied that bacterial PMF may serve as a novel antibacterial target for future antibiotic discovery and development. Furthermore, consistent with previously reported bactericidal AMPs such as WRK-12 and SLAP-S25 [55,56], ROS production was also evidenced to be important for the antibacterial activity of WW307.

In conclusion, we revealed that amphipathic peptide WW307 is a bacterial membrane-active AMP that could simultaneously disrupt the permeability of bacterial membrane, dissipate membrane potential and induce the production of ROS. These findings suggested that WW307 is a potent antibiotic candidate and shows great potential for clinical use to combat MDR pathogens.

## Figures and Tables

**Table 1:** Key physicochemical parameters of amphipathic peptides.

Name	Sequence (*N* → *C*)	Formula	MW	Net Charge	pI ^#^	Purity (%)
WW291	WWWLRKIW-NH_2_	C_68_H_89_N_17_O_8_	1272.58	+2	11.00	96.57%
WW304	WWWLRRRW-NH_2_	C_68_H_90_N_22_O_8_	1343.62	+3	12.30	95.33%
WW295	RKIWWWWL-NH_2_	C_68_H_89_N_17_O_8_	1272.58	+2	11.00	95.49%
WW307	RRRWWWWV-NH_2_	C_67_H_88_N_22_O_8_	1329.59	+3	12.30	95.79%

^#^ The pI values of derivatives were determined by ExPASy (http://web.expasy.org/compute_pi/, accessed on 15 March 2021).

**Table 2:** Antimicrobial activity of amphiphilic peptides against a panel of MDR pathogenic bacteria (MIC, μg/mL).

Organisms and Phenotypes	WW304	WW307	Melittin	MEM	COL	TIG
*S. aureus* G16 (RIF^R^)	8	1	2	2	1	<0.0625
MRSA T144	8	4	4	1	16	0.25
*E*. *coli* B2 (*mcr-1* + *bla*_NDM-5_)	64	4	8	32	8	2
*E*. *coli* C3 (*bla*_NDM-1_)	64	4	8	8	<0.125	2
*E*. *coli* G6 (*bla*_NDM-5_)	64	8	4	64	0.5	2
*E*. *coli* G92 (*mcr-1*)	64	4	4	<0.125	4	4
*E*. *coli* CP131 (*mcr-3*)	32	4	4	<0.125	4	2
*E*. *coli* 1F28 (*tet*(X4))	32	4	4	0.25	0.125	16
*A. baumannii* C222 (*tet*(X6))	64	4	8	<0.125	<0.125	64
*P*. *cibarius* HNCF44W (*bla*_NDM-1_ + *tet*(X6))	64	2	4	>16	>256	64

RIF^R^, rifampicin-resistant; MRSA, methicillin-resistant *Staphylococcus aureus*. MEM, meropenem; COL, colistin; TIG, tigecycline.

**Table 3:** Thermal, pH, salts and protease stability of WW307 against MRSA T144 and *E. coli* B2 (MIC, μg/mL).

Treatments	WW307	
	MRSA T144	*E. coli* B2
Control	4	4
Temperature		
40 °C	4	4
60 °C	4	4
80 °C	4	4
100 °C	4	4
121 °C	4	4
pH		
2	4	4
4	4	4
6	4	4
8	4	4
10	4	4
12	4	8
Salt ions (10 mM)		
Na^+^	4	4
K^+^	4	4
Mg^2+^	16	16
Protease (1 mg/mL)		
Pepsin	4	4
Trypsin	>128	>128
Papain	>128	>128
Serum (10%)	8	8
DMEM (10%)	8	8

## Data Availability

All data in this study have been included in this manuscript.
